# Nongenotoxic effects and a reduction of the DXR-induced genotoxic effects of *Helianthus annuus* Linné (sunflower) seeds revealed by micronucleus assays in mouse bone marrow

**DOI:** 10.1186/1472-6882-14-121

**Published:** 2014-04-02

**Authors:** Marcelo Fabiano Gomes Boriollo, Luiz Silva Souza, Marielly Reis Resende, Thaísla Andrielle da Silva, Nelma de Mello Silva Oliveira, Maria Cristina Costa Resck, Carlos Tadeu dos Santos Dias, João Evangelista Fiorini

**Affiliations:** 1 Laboratório de Farmacogenômica e Biologia Molecular, Faculdade de Ciências Médicas & Centro de Pesquisa e Pós–graduação, Universidade José do Rosário Vellano (UNIFENAS), Campus Universitário, Rod. MG 179, Km 0, Alfenas, MG CEP: 37130-000, Brasil; 2 Centro de Pesquisa e Pós–graduação em Ciência Animal, Área de Patologia e Farmacologia Animal, Universidade José do Rosário Vellano (UNIFENAS), Alfenas, Minas Gerais, Brasil; 3 Centro de Cirurgia Experimental e Farmacologia, Universidade Estadual de Campinas, Campinas (UNICAMP), São Paulo, Brasil; 4 Laboratório de Ecotoxicologia e Microbiologia Ambiental, Faculdade de Tecnologia, Universidade Estadual de Campinas (UNICAMP), Limeira, São Paulo, Brasil; 5 Departamento de Ciências Exatas, Escola de Agricultura “Luiz de Queiroz”, Universidade de São Paulo (ESALQ/USP), Piracicaba, SP, Brasil

**Keywords:** Bone marrow, *Helianthus annuus* L. (sunflower), Micronucleus assay, Rodents, Tincture, Oil

## Abstract

**Background:**

This research evaluated the genotoxicity of oil and tincture of *H. annuus* L. seeds using the micronucleus assay in bone marrow of mice. The interaction between these preparations and the genotoxic effects of doxorubicin (DXR) was also analysed (antigenotoxicity test).

**Methods:**

Experimental groups were evaluated at 24-48 h post treatment with N-Nitroso-N-ethylurea (positive control – NEU), DXR (chemotherapeutic), NaCl (negative control), a sunflower tincture (THALS) and two sources of sunflower oils (POHALS and FOHALS). Antigenotoxic assays were carried out using the sunflower tincture and oils separately and in combination with NUE or DXR.

**Results:**

For THALS, analysis of the MNPCEs showed no significant differences between treatment doses (250–2,000 mg.Kg^-1^) and NaCl. A significant reduction in MNPCE was observed when THALS (2,000 mg.Kg^-1^) was administered in combination with DXR (5 mg.Kg^-1^). For POHALS or FOHALS, analysis of the MNPCEs also showed no significant differences between treatment doses (250–2,000 mg.Kg^-1^) and NaCl. However, the combination DXR + POHALS (2,000 mg.Kg^-1^) or DXR + FOHALS (2,000 mg.Kg^-1^) not contributed to the MNPCEs reduction.

**Conclusions:**

This research suggests absence of genotoxicity of THALS, dose-, time- and sex-independent, and its combination with DXR can reduce the genotoxic effects of DXR. POHALS and FOHALS also showed absence of genotoxicity, but their association with DXR showed no antigenotoxic effects.

## Background

The cultivated sunflower (*Helianthus annuus* L.) is one of 67 species in the genus *Helianthus*. It is a dicotyledonous plant and a member of the *Compositae* (*Asteraceae*) family, having a typical composite flower [1]. The composition of the seed is markedly affected by the sunflower variety [2,3]. Nevertheless, the composition ranges of sunflower dehulled seeds (on a percentage dry weight basis) is as follows [4]: protein ^20.4–40.0%^; peptides, amino acids and other non–protein nitrogen ^1–13%^; carbohydrates ^4–10%^; lipids ^47–65%^; fatty acids (palmitic acid ^5–7%^, atearic ^2–6%^; arachidic acid ^0.0–0.3%^, oleic acid ^15–37%^; linoleic acid ^51–73%^, and linolenic acid ^*<*0*.*3%^); tocopherol ^0.07%^; carotenoids ^0.01–0.02%^; vitamin B1 ^0.002%^; chlorogenic acid (CGA) ^0.5–2.4%^; quinic acid (QA) ^0.12–0.25%^; caffeic acid (CA) ^0.05–0.29%^; total minerals ^3–4%^; potassium ^0.67–0.75%^; phosphorus ^0.60–0.94%^; sulphur ^0.26–0.32%^; magnesium ^0.35–0.41%^; calcium ^0.08–0.10%^; and sodium ^0.02%^.

Tocopherols are excellent natural antioxidants that protect oils against oxidative rancidity. The α form has the highest biological vitamin E activity, and the γ form has been reported to have the highest antioxidant activity [5]. The sterols found in sunflower oils include β-sitosterol, stigmasterol, campesterol, δ-5-avenasterol, and δ-7-stigmasterol [6,7]. Plant sterols are only minimally absorbed by humans, and their ingestion appears to inhibit intestinal cholesterol and bile acid absorption [8]. Most trace metals in refined, bleached and deodorized sunflower seed oil are removed during processing. It is particularly important that copper and iron be removed because these metals greatly reduce the oxidative stability of the oil [9]. Other metals, such as lead and cadmium, are of particular concern due to their toxicity and their supposed link to coronary heart disease and hypertension [10].

In drug development, the genotoxicity assays represent a considerable effort, as most pharmaceutical organizations evaluate a new therapeutic agent based on *in vitro* and *in vivo* data genotoxic [11]. In this context, tests to evaluate the genotoxic activity of the plants used by the population as well as their isolated compounds are necessary and important for establishing control measures in widespread use. Furthermore, it is necessary to clarify the mechanisms and conditions that mediate the proposed biological effect before plants are considered as therapeutic agents [12]. As far as genotoxicity studies are concerned, the *in vivo* micronucleus (MN) assay in rodent bone marrow plays a crucial role in the test battery aimed at identifying hazardous mutagens [13]; this assay is especially suited to assessing genotoxic hazards because it allows consideration of multiple factors, such as *in vivo* metabolism, pharmacokinetics and DNA repair processes, even though these processes vary among species, among tissues and among genetic endpoints [14-17]. In addition, understanding the genotoxic effects induced by phytotherapeutics and foods employing the mammalian *in vivo* MN assay has been the goal of several researchers groups [18-20].

In order to contribute to the information on the genotoxic potential of herbal extracts and food, the present study evaluated the genotoxic effects of two sources of oil and tincture of *H. annuus* L. (sunflower) seeds using *in vivo* micronucleus assays in mouse bone marrow. The effect of the maximum permissible concentration of *H. annuus* L. (oils and tincture) on the doxorubicin (DXR)–induced genotoxic effects in mice bone marrow was also studied (i.e., antigenotoxicity assay).

## Methods

### Phytotherapeutics

Tincture and oil of sunflower seeds were purchased commercially and stored according to the manufacturer's recommendations [tincture of *H. annuus* L. seeds (THALS) – Yod Comércio de Produtos Naturais Ltda., cat. # 544606, Campinas, SP, Brazil; pharmaceutical oil of *H. annuus* L. seeds (POHALS) – Farmácia de Manipulação Alfenense Ltda., Alfenas, MG, Brazil; food oil of *H. annuus* L. seeds (FOHALS) – Agricultural Cargill S.A., Mairinque, SP, Brazil]. Aliquots (1.5 L) of this tincture were submitted to solvent removal proceedings by rotary evaporation (40 rpm) (Rotavapor Model R-215) coupled in bath heating systems ^50–60°C^ (Bath Heating model B-491), vacuum pump ^500 mmHg^ (Vacuum Pump V-700 with Automatic Vacuum Controller V-855), recirculator (Recirculator Chiller F-100) and evaporation bottle (Büchi Labortechnik AG, Switzerland). The final product was transferred to a reaction bottle 1 L (SCHOTT^®^ DURAN^®^) and kept at -20°C for 24 hours in order to evaluate the freezing of the final product and the efficacy of the solvent evaporation process [21]. Then, aliquots (40 mL) of this final product was transferred into glass vials penicillin type (50 mL) and lyophilized (Lyophilizer model Alpha 1–2 LDPlus, Martin Christ Gefriertrocknungsanlagen GmbH^©^, Germany) and their dry mass were measured (Electronic Analytical Balance AUW-220D, Shimadzu Corp., Kyoto, Japan). The lyophilized final product was prepared in aqueous solvent (150 mM NaCl in water type 1) at concentrations of 2×, sterilized by filtration (Millipore Corporation, hydrophilic Durapore^®^ PVDF, 0.22 μm, ∅ 47 mm, cat. # GVWP 047 00), and stored in sterile polypropylene tubes (50 mL) at -70°C until moment of use.

### System – test *in vivo*

Healthy, heterogeneous, young adult male and female *Swiss albinus* (Unib: SW) mice (between 7 and 12 weeks – pubescent period), with a body weight between 30 g and 40 g (i.e., the variation weight between the animals, for each sex, should not exceed the ± 20% of medium mass) were provided by CEMIB (*Centro Multidisciplinar para Investigação Biológica na Área da Ciência em Animais de Laboratório* – UNICAMP; http://www.cemib.unicamp.br/), and erythrocytes from the bone marrow of these mice were used in the micronucleus assay [14,17,22]. The animals were kept in groups of the same sex, in polypropylene boxes, in an air–conditioned environment to 22°C ± 3°C, with relative air humidity of 50% ± 20%, and with 12–hour day–night cycles (i.e., 12 h light and 12 h dark). These were fed with Purina^®^ Labina commercial rations (Nestlé Purina Pet Care Company) and water *ad libitum*, and acclimated to laboratory conditions for 7 days (a trial period) before the execution of the experiment. At the end of the trial period, each animal was weighed and, according to the weight, received 2 mL/100 g body weight of the indicated liquid (negative control, positive control, chemotherapeutic and phytotherapeutic). After the experimental treatment, the animals were euthanized by CO_2_ asphyxiation in adapted acrylic chambers [14]. This research was approved by Committee of Ethics in Research Involving Animals of UNIFENAS (CEPEAU Protocol No. 04A/2008).

### Experimental groups

Groups of animals (consisting of 3 males and 3 females each) were treated using a single dosing regimen administered by gavage (phytotherapeutic and negative control) or intraperitoneally (chemotherapeutic and positive control) and two euthanasia times (24 and 48 h), based on a regulatory recommendation regarding the *in vivo* micronucleus assay [14,17]:

▪ Control groups: 150 mM NaCl (negative control), 50 mg.Kg^-1^ of N-Nitroso-N-ethylurea (positive control: NEU, Sigma N8509, CAS no. 759-73-9) and 5 mg.Kg^-1^ of doxorubicin hydrochloride [20] (chemotherapeutic: DXR, Eurofarma Laboratórios Ltda., CAS no. 23214-92-8).

▪ Genotoxicity test (phytotherapeutics): THALS (250–2,000 mg.Kg^-1^), POHALS (250–2,000 mg.Kg^-1^) and FOHALS (250–2,000 mg.Kg^-1^). The maximum tolerated dose (MTD) was defined as (*i*) the highest dose that can be administered without inducing lethality or excessive toxicity during the study causing moribund euthanasia, or (*ii*) a dose that produces some indication of toxicity of the bone marrow (e.g. a reduction in the proportion of immature erythrocytes among total erythrocytes in the bone marrow), or (*iii*) 2,000 mg.Kg^-1^[14,17].

▪ Antigenotoxicity test 1 (phytotherapeutics + chemotherapeutic) [20]: THALS (2,000 mg.Kg^-1^) + DXR (5 mg.Kg^-1^), FOHALS (2,000 mg.Kg^-1^) + DXR (5 mg.Kg^-1^) and FOHALS (2,000 mg.Kg^-1^) + DXR (5 mg.Kg^-1^).

▪ Antigenotoxicity test 2 (phytotherapeutics + positive control): THALS (2,000 mg.Kg^-1^) + NEU (50 mg.Kg^-1^), POHALS (2,000 mg.Kg^-1^) + NEU (50 mg.Kg^-1^) and POHALS (2,000 mg.Kg^-1^) + NEU (50 mg.Kg^-1^).

### Processing the bone marrow and cell analysis

Shortly after euthanasia, the femora were surgically and aseptically removed, and the animals appropriately discarded. Each femur was sectioned at the proximal end and the contents of the spinal canal were washed with 1.5 mL of 150 mM NaCl solution and transferred to a 15 mL centrifuge tube [14,17,23]. This material was resuspended with a Pasteur pipette to ensure a random distribution of bone marrow cells. The suspension was then centrifuged at 1,000 rpm (*Centrífuga de Bancada Microprocessada*, Mod. NT 810, Nova Técnica Ind. e Com. de Equip. para Laboratório Ltda., Piracicaba, SP, Brazil) for 5 minutes. The supernatant was discarded and the resulting sediment was resuspended in 500 μL of 150 mM NaCI solution added 4% formaldehyde. The slides were prepared by smearing (2 slides per animal), dried at room temperature for 24 h and stained with Leishman's eosin methylene blue dye [pure dye for 3 min, followed by diluted dye in water type 1 (1:6) for 15 min] to differentiate polychromatic erythrocyte (PCE) from normochromatic erythrocyte (NCE).

Polychromatic erythrocytes (PCEs) were observed at a magnification of 1000× using optical microscopy (Nikon Eclipse E–200), counted (at least 2000 polychromatic erythrocytes anucleated per animal were scored for the incidence of micronucleated polychromatic erythrocytes) with the aid of a digital cell counter (Contador Diferencial CCS02, Kacil Indústria e Comércio Ltda., PE, Brasil Contador Diferencial CCS02, Kacil Indústria e Comércio Ltda., PE, Brazil) and photographed using an 8.1 Megapixel Digital Camera (DC FWL 150). The number of PCEs and NCEs, the number and frequency of micronucleated polychromatic erythrocytes (MNPCEs) were reported. In order to evaluate bone-marrow toxicity, the ratio of PCE to NCE was also observed [14,17]. This PCE/NCE ratio is an indicator of the acceleration or inhibition of erythropoiesis and it has been reported to vary with scoring time. A continuous decline in the PCE/NCE ratio may be due to the inhibition of cell division, the killing of erythroblasts, the removal of damaged cells, or dilution of the existing cell pool with newly formed cells [20].

### Statistical analysis

The data obtained in the micronucleus assay were submitted to *one–way* analysis of variance (ANOVA), using a factorial scheme of 10 × 2 × 2 (treatment × sex × euthanasia time), and medium comparison with Tukey's test (α = 0.05) using SAS^®^ version 9.2 computer software.

## Results and discussion

*H. annuus* L. has been considered an important source of natural oil for centuries and has been used as a preventive medicine against diuresis, diarrhoea, and various inflammatory diseases [24], and has also been used for the relief of asthmatic symptoms [25], gastric protection [26,27], its healing properties [28], anti-inflammatory action [29] and antimicrobial properties [26,28]. However, studies aimed at understanding the genotoxic and mutagenic effects of *H. annuus* L. were subject of comparatively little research [19,30], which drove us to evaluate the harmful genotoxic and antigenotoxic properties (i.e., clastogenicity and/or aneugenicity) of oil and tincture of *H. annuus* L. seeds using the MN assay *in vivo*.

The numbers and frequencies of MNPCEs and the PCE/NCE ratio in the bone marrow of mice were analyzed statistically for each one of the animal groups treated with only tincture (THALS) or oils (POHALS or FOHALS) of sunflower seeds – genotoxic assays – and for each one of the groups treated with phytotherapeutics and chemotherapeutic agent DXR (THALS + DXR, POHALS + DXR or FOHALS + DXR) – antigenotoxic assays –, as well as control groups.

For animal groups treated with THALS, analysis of the MNPCEs showed no significant differences (*p* < 0.05) between all the treatment doses (250–2,000 mg.Kg^-1^) and negative control (NaCl). These results suggest absence of genotoxicity of THALS, regardless of the dose of phytotherapic administration (250–2,000 mg.Kg^-1^), the treatment time (24 and 48 h) or the sex of the animal (male and female). Treatment of mice with 5 mg.Kg^-1^ DXR significantly induced MNPCE at 24 and 48 h post treatment and for both sexes, whose MNPCE frequencies were significantly above (*p* < 0.05) those observed in the positive NEU control (50 mg.Kg^-1^). However, the reduction in MNPCE (*p* < 0.05) was observed when THALS (2,000 mg.Kg^-1^) is administered in combination with the chemotherapy agent DXR (5 mg.Kg^-1^), suggesting antigenotoxic effects (anticlastogeny and/or antianeugeny). Therefore, THALS provides a partial protection against the genotoxic effects induced by DXR in the bone marrow of mice, regardless of the treatment time (24 and 48 h) or the sex of the animal, although the genotoxic effect observed in this treatment combination has is similar (i.e., numbers and frequencies of MNPCEs) to that observed in NEU-treated animals. The analysis obtained from the PCE/NCE ratio showed no significant differences (*p* < 0.05) between all doses of THALS (250–2,000 mg.Kg^-1^), THALS (2,000 mg.Kg^-1^) + DXR (5 mg.Kg^-1^) and negative controls. These results suggest that there is not systemic toxicity of THALS and/or DXR under the MN assay conditions, regardless of the phytotherapeutic doses and times, but sex-dependent (Table 1).

**Table 1:** **The incidence of MNPCEs and PCE/NCE ratio in bone marrow of male and female *Swiss albinus *** **mice after testing for 24 h and 48 h**

**Treatment**	**Number of PCEs analyzed**		**PCEMNs**				**PCE/(PCE + NCE)**		**NCE ( *n *)**	
	**24 h**	**48 h**	**24 h**	**48 h**	**24 h**	**48 h**	**24 h **^**A”**^	**48 h **^**B”**^	**24 h**	**48 h**
			**( *n *) **^**A**^	**( *n *) **^**A**^	**(%) **^**A’**^	**(%) **^**A’**^				
*150 mM NaCl*										
♀_1_	2095	2097	7	10	0.33	0.48	1.00	1.00	5	3
♀_2_	2094	2095	9	10	0.43	0.48	1.00	1.00	6	5
♀_3_	2087	2089	11	8	0.53	0.38	0.99	0.99	13	11
Σ ♀	Σ 6276	Σ 6281	Σ 27	Σ 28	0.43 ±0.10	0.45 ±0.05	1.00 ±0.00	1.00 ±0.00	Σ 24	Σ 19
♂_1_	2095	2088	9	13	0.43	0.62	1.00	0.99	5	12
♂_2_	2055	2088	12	11	0.58	0.53	0.98	0.99	45	12
♂_3_	2058	2084	7	11	0.34	0.53	0.98	0.99	42	16
Σ ♂	Σ 6208	Σ 6260	Σ 28	Σ 35	0.45 ±0.12	0.56 ±0.06	0.99 ±0.01	0.99 ± 0.00	Σ 92	Σ 40
Σ ♂ and ♀	Σ 12484	Σ 12541	Σ 55 ^A^	Σ 63 ^A^	0.44 ±0.08 ^A’^	0.50 ±0.06 ^A’^	0.99 ±0.01 ^A”^	1.00 ±0.00 ^A”^	Σ 116	Σ 59
*N–Nitroso–N–ethylurea – NEU (50 mg.Kg*^*-1*^*)*										
♀_1_	2148	2075	38	36	1.77	1.73	0.49	0.65	2252	1125
♀_2_	1884	2032	32	34	1.70	1.67	0.54	0.81	1616	468
♀_3_	2002	1948	15	31	0.75	1.59	0.61	0.93	1298	152
Σ ♀	Σ 6034	Σ 6055	Σ 85	Σ 101	1.41 ±0.57	1.67 ±0.07	0.54 ±0.06	0.80 ±0.14	Σ 5166	Σ 1745
♂_1_	2025	1999	64	31	3.16	1.55	0.41	0.36	2875	3501
♂_2_	2028	1916	105	40	5.18	2.09	0.51	0.55	1972	1584
♂_3_	2004	2069	25	38	1.25	1.84	0.67	0.65	996	1131
Σ ♂	Σ 6057	Σ 5984	Σ 194	Σ 109	3.20 ±1.97	1.83 ±0.27	0.53 ±0.13	0.52 ±0.14	Σ 5843	Σ 6216
Σ ♂ and ♀	Σ 12091	Σ 12039	Σ 279 ^B^	Σ 210 ^B^	2.30 ±1.66 ^B’^	1.75 ±0.18 ^B’^	0.54 ±0.06 ^B”^	0.66 ±0.16 ^B”^	Σ 11009	Σ 7961
*Doxorubicin hydrochloride – DXR (5 mg.Kg*^*-1*^*)*										
♀_1_	2091	2017	49	36	2.34	1.78	0.72	0.96	809	83
♀_2_	2106	2077	73	63	3.47	3.03	0.98	0.99	44	23
♀_3_	2056	2092	57	50	2.77	2.39	0.84	0.95	394	108
Σ ♀	Σ 6253	Σ 6186	Σ 179	Σ 149	2.86 ±0.57	2.40 ±0.62	0.85 ±0.13	0.97 ±0.02	Σ 1247	Σ 214
♂_1_	2067	2086	53	61	2.56	2.92	0.98	0.95	33	114
♂_2_	2063	2042	56	70	2.71	3.43	0.98	0.97	37	58
♂_3_	2082	2075	46	50	2.21	2.41	0.99	0.99	18	25
Σ ♂	Σ 6212	Σ 6203	Σ 155	Σ 181	2.50 ±0.26	2.92 ±0.51	0.99 ±0.00	0.97 ±0.02	Σ 88	Σ 197
Σ ♂ and ♀	Σ 12465	Σ 12389	Σ 334^C^	330^C^	2.68 ±0.42 ^C’^	2.66 ±0.43 ^C’^	0.92 ±0.07 ^A”^	0.97 ±0.01 ^A”^	Σ 1335	Σ 411
*THALS – Tincture of H. annuus L. seeds (250 mg.Kg*^*-1*^*)*										
♀_1_	2097	2192	8	7	0.38	0.32	0.99	0.99	14	12
♀_2_	2105	2057	7	12	0.33	0.58	0.99	0.99	31	28
♀_3_	2181	2092	12	12	0.55	0.57	0.99	1.00	19	8
Σ ♀ ^A A^	Σ 6383	Σ 6341	Σ 27	Σ 31	0.42 ±0.11	0.49 ±0.15	0.99 ±0.00	0.99 ±0.01	Σ 64	Σ 48
♂_1_	2041	2070	7	7	0.34	0.34	0.99	1.00	27	10
♂_2_	2050	2062	9	6	0.44	0.29	0.99	0.99	17	19
♂_3_	2055	2065	10	12	0.49	0.58	0.99	0.99	12	15
Σ ♂ ^A A^	Σ 6146	Σ 6197	Σ 26	Σ 25	0.42 ±0.07	0.40 ±0.16	0.99 ±0.00	0.99 ±0.00	Σ 56	Σ 44
Σ ♂ and ♀	Σ 12529	Σ 12538	Σ 53 ^A^	Σ 56 ^A^	0.42 ±0.09 ^A’^	0.45 ±0.14 ^A’^	0.99 ±0.00 ^A”^	0.99 ±0.00 ^A”^	Σ 120	Σ 92
*THALS – Tincture of H. annuus L. seeds (500 mg.Kg*^*-1*^*)*										
♀_1_	2086	2146	12	16	0.58	0.75	0.99	0.99	17	22
♀_2_	2078	2060	12	13	0.58	0.63	0.99	0.99	25	11
♀_3_	2072	2046	13	11	0.63	0.54	0.99	0.99	30	26
Σ ♀ ^A A^	Σ 6236	Σ 6252	Σ 37	Σ 40	0.59 ±0.03	0.64 ±0.10	0.99 ±0.00	0.99 ±0.00	Σ 72	Σ 59
♂_1_	2071	2075	17	8	0.82	0.39	0.98	0.99	32	13
♂_2_	2074	2081	12	11	0.58	0.53	0.99	0.99	29	20
♂_3_	2072	2067	11	10	0.53	0.48	0.99	0.99	22	11
Σ ♂ ^A A^	Σ 6217	Σ 6223	Σ 40	Σ 29	0.64 ±0.16	0.47 ±0.07	0.99 ±0.00	0.99 ±0.00	Σ 83	Σ 44
Σ ♂ and ♀	Σ 12453	Σ 12475	Σ 77 ^A^	Σ 69 ^A^	0.62 ±0.10 ^A’^	0.55 ±0.12 ^A’^	0.99 ±0.00 ^A”^	0.99 ±0.00 ^A”^	Σ 155	Σ 103
*THALS – Tincture of H. annuus L. seeds (1,000 mg.Kg*^*-1*^*)*										
♀_1_	2083	2165	13	17	0.62	0.79	0.98	0.99	35	17
♀_2_	2075	2076	12	14	0.58	0.67	0.99	0.99	24	25
♀_3_	2070	2061	13	14	0.63	0.68	0.98	0.98	49	34
Σ ♀ ^A A^	Σ 6228	Σ 6302	Σ 38	Σ 45	0.61 ±0.03	0.71 ±0.06	0.98 ±0.01	0.99 ±0.00	Σ 108	Σ 76
♂_1_	2079	2084	18	10	0.87	0.48	0.98	0.99	32	31
♂_2_	2092	2095	13	11	0.62	0.53	0.99	0.99	30	21
♂_3_	2073	2077	11	8	0.53	0.39	0.98	0.98	32	40
Σ ♂ ^A A^	Σ 6244	Σ 6256	Σ 42	Σ 29	0.67 ±0.17	0.46 ±0.07	0.99 ±0.00	0.99 ±0.00	Σ 94	Σ 92
Σ ♂ and ♀	Σ 12472	Σ 12558	Σ 80 ^A^	Σ 74 ^A^	0.64 ±0.12 ^A’^	0.59 ±0.15 ^A’^	0.98 ±0.00 ^A”^	0.99 ±0.00 ^A”^	Σ 202	Σ 168
*THALS – Tincture of H. annuus L. seeds (1,500 mg.Kg*^*-1*^*)*										
♀_1_	2057	2171	13	17	0.63	0.78	0.98	0.98	42	39
♀_2_	2061	2063	14	18	0.68	0.87	0.99	0.98	31	36
♀_3_	2026	2090	10	11	0.49	0.53	0.98	0.99	44	23
Σ ♀ ^A A^	Σ 6144	Σ 6324	Σ 37	Σ 46	0.60 ±0.10	0.73 ±0.18	0.98 ±0.00	0.98 ±0.00	Σ 117	Σ 98
♂_1_	2075	2048	14	12	0.67	0.59	0.98	0.98	45	48
♂_2_	2063	2076	13	8	0.63	0.39	0.97	0.99	58	24
♂_3_	2068	2079	17	15	0.82	0.72	0.98	0.99	41	31
Σ ♂ ^A A^	Σ 6206	Σ 6203	Σ 44	Σ 35	0.71 ±0.10	0.56 ±0.17	0.98 ±0.00	0.98 ±0.01	Σ 144	Σ 103
Σ ♂ and ♀	Σ 12350	Σ 12527	Σ 81 ^A^	Σ 81 ^A^	0.66 ±0.11 ^A’^	0.65 ±0.18 ^A’^	0.98 ±0.00 ^A”^	0.98 ±0.00 ^A”^	Σ 261	Σ 201
*THALS – Tincture of H. annuus L. seeds (2,000 mg.kg*^*-1*^*)*										
♀_1_	2055	2061	15	14	0.73	0.68	0.97	0.98	59	39
♀_2_	2052	2060	17	19	0.83	0.92	0.97	0.98	62	40
♀_3_	2079	2061	15	15	0.72	0.73	0.98	0.98	35	39
Σ ♀ ^A A^	Σ 6186	Σ 6182	Σ 47	Σ 48	0.76 ±0.06	0.78 ±0.13	0.98 ±0.01	0.98 ±0.00	Σ 156	Σ 118
♂_1_	2145	2071	22	10	1.03	0.48	0.97	0.99	58	29
♂_2_	2064	2028	8	12	0.39	0.59	0.98	0.97	40	72
♂_3_	2047	2071	18	15	0.88	0.72	0.97	0.99	56	29
Σ ♂ ^A A^	Σ 6256	Σ 6170	Σ 48	Σ 37	0.76 ±0.33	0.60 ±0.12	0.98 ±0.00	0.98 ±0.01	Σ 154	Σ 130
Σ ♂ and ♀	Σ 12442	Σ 12352	Σ 95 ^A^	Σ 85 ^A^	0.76 ±0.21 ^A’^	0.69 ±0.15 ^A’^	0.98 ±0.01 ^A”^	0.98 ±0.01 ^A”^	Σ 310	Σ 248
*THALS (2 g.kg*^*-1*^*) + NEU (50 mg.Kg*^*-1*^*)*										
♀_1_	2074	2048	27	32	1.30	1.56	0.99	0.85	26	352
♀_2_	2070	2076	30	27	1.45	1.30	0.99	0.99	30	24
♀_3_	2079	2083	32	33	1.54	1.58	0.99	0.99	21	17
Σ ♀ ^A A^	Σ 6223	Σ 6207	Σ 89	Σ 92	1.43 ±0.12	1.48 ±0.16	0.99 ±0.00	0.94 ±0.08	Σ 77	Σ 393
♂_1_	2077	2076	35	37	1.69	1.78	0.99	0.99	23	24
♂_2_	2075	2078	36	35	1.73	1.68	0.99	0.99	25	22
♂_3_	2077	2074	32	37	1.54	1.78	0.99	0.99	23	26
Σ ♂ ^A A^	Σ 6229	Σ 6228	Σ 103	Σ 109	1.65 ±0.10	1.75 ±0.06	0.99 ±0.00	0.99 ±0.00	Σ 71	Σ 72
Σ ♂ and ♀	Σ 12452	Σ 12435	Σ 192 ^B^	Σ 201 ^B^	1.54 ±0.16 ^B’^	1.62 ±0.18 ^B’^	0.99 ±0.00 ^A”^	0.97 ±0.06 ^A”^	Σ 148	Σ 465
*THALS (2 g.kg*^*-1*^*) + DXR (5 mg.Kg*^*-1*^*)*										
♀_1_	2074	2075	36	38	1.74	1.83	0.99	0.99	26	25
♀_2_	2075	2079	36	29	1.73	1.39	0.99	0.99	25	21
♀_3_	2074	2082	34	36	1.64	1.73	0.99	0.99	26	18
Σ ♀ ^A A^	Σ 6223	Σ 6236	Σ 106	Σ 103	1.70 ±0.06	1.65 ±0.23	0.99 ±0.00	0.99 ±0.00	Σ 77	Σ 64
♂_1_	2080	2089	34	28	1.63	1.34	0.99	0.99	20	11
♂_2_	2081	2077	30	34	1.44	1.64	0.99	0.99	19	23
♂_3_	2090	2082	33	34	1.58	1.63	1.00	0.99	10	18
Σ ♂ ^A A^	Σ 6251	Σ 6248	Σ 97	Σ 96	1.55 ±0.10	1.54 ±0.17	0.99 ±0.00	0.99 ±0.00	Σ 49	Σ 52
Σ ♂ and ♀	Σ 12474	Σ 12484	Σ 203 ^B^	Σ 199 ^B^	1.63 ±0.11 ^B’^	1.59 ±0.19 ^B’^	0.99 ±0.00 ^A”^	0.99 ±0.00 ^A”^	Σ 126	Σ 116

Means with the same letter are not significantly different (*p* < 0.05).

Shown are data from the controls (NaCl, NEU and DXR), an evaluation of the genotoxicity of THALS, and an evaluation of the antigenotoxicity of THALS (THALS + NEU and THALS + DXR).

For animal groups treated with POHALS (Table 2) or FOHALS (Table 3), analysis of the MNPCEs showed no significant differences (*p* < 0.05) between all the treatment doses (250–2,000 mg.Kg^-1^) and negative control (NaCl). These results suggest absence of genotoxicity for both sources of sunflower oil (pharmaceutical and food), regardless of the dose of oil administration (250–2,000 mg.Kg^-1^) or treatment time (24 and 48 h), but it was sex-dependent. Treatment of mice with DXR (5 mg.Kg^-1^) + POHALS (2,000 mg.Kg^-1^) or DXR (5 mg.Kg^-1^) + FOHALS (2,000 mg.Kg^-1^) not contribute to the MNPCEs reduction at 24 and 48 h post treatment and for both sexes, suggesting that both sources of sunflower oil not decrease the DXR-induced genotoxic effects and therefore they do not have antigenotoxic effects (anticlastogeny and/or antianeugeny). The analysis obtained from the PCE/NCE ratio showed no significant differences (*p* < 0.05) between all doses of POHALS (250–2,000 mg.Kg^-1^) and negative controls, time-dependent and sex-independent. For FOHALS, the PCE/NCE ratio showed significant differences (*p* < 0.05) only in the highest dose (2,000 mg.Kg^-1^) tested, time-independent and sex-dependent. These results suggest that the systemic toxicity of sunflower oil can be dependent on its source and its highest dose used. In addition, treatments with DXR (5 mg.Kg^-1^) + POHALS (2,000 mg.Kg^-1^) or DXR (5 mg.Kg^-1^) + FOHALS (2,000 mg.Kg^-1^) significantly decrease the PCE/NCE ratio in mouse bone marrow. These results suggests that the association sunflower oil and chemotherapeutic agent DXR can potentize the systemic toxicity, regardless of the sex (only POHALS) and time (only FOHALS).

**Table 2:** **The incidence of MNPCEs and PCE/NCE ratio in bone marrow of male and female *Swiss albinus *** **mice after testing for 24 h and 48 h**

**Treatment**	**Number of PCEs analyzed**		**PCEMNs**				**PCE/(PCE + NCE)**		**NCE ( *n *)**	
	**24 h**	**48 h**	**24 h**	**48 h**	**24 h**	**48 h**	**24 h **^**A”**^	**48 h **^**B”**^	**24 h**	**48 h**
			**( *n *) **^**A**^	**( *n *) **^**A**^	**(%) **^**A’**^	**(%) **^**A’**^				
*150 mM NaCl*										
♀_1_	2095	2097	7	10	0.33	0.48	1.00	1.00	5	3
♀_2_	2094	2095	9	10	0.43	0.48	1.00	1.00	6	5
♀_3_	2087	2089	11	8	0.53	0.38	0.99	0.99	13	11
Σ ♀	Σ 6276	Σ 6281	Σ 27	Σ 28	0.43 ±0.10	0.45 ±0.05	1.00 ±0.00	1.00 ±0.00	Σ 24	Σ 19
♂_1_	2095	2088	9	13	0.43	0.62	1.00	0.99	5	12
♂_2_	2055	2088	12	11	0.58	0.53	0.98	0.99	45	12
♂_3_	2058	2084	7	11	0.34	0.53	0.98	0.99	42	16
Σ ♂	Σ 6208	Σ 6260	Σ 28	Σ 35	0.45 ±0.12	0.56 ±0.06	0.99 ±0.01	0.99 ± 0.00	Σ 92	Σ 40
Σ ♂ and ♀	Σ 12484	Σ 12541	Σ 55 ^A^	Σ 63 ^A^	0.44 ±0.08 ^A’^	0.50 ±0.06 ^A’^	0.99 ±0.01 ^A”^	1.00 ±0.00 ^A”^	Σ 116	Σ 59
*N–Nitroso–N–ethylurea – NEU (50 mg.Kg*^*–1*^*)*										
♀_1_	2148	2075	38	36	1.77	1.73	0.49	0.65	2252	1125
♀_2_	1884	2032	32	34	1.70	1.67	0.54	0.81	1616	468
♀_3_	2002	1948	15	31	0.75	1.59	0.61	0.93	1298	152
Σ ♀	Σ 6034	Σ 6055	Σ 85	Σ 101	1.41 ±0.57	1.67 ±0.07	0.54 ±0.06	0.80 ±0.14	Σ 5166	Σ 1745
♂_1_	2025	1999	64	31	3.16	1.55	0.41	0.36	2875	3501
♂_2_	2028	1916	105	40	5.18	2.09	0.51	0.55	1972	1584
♂_3_	2004	2069	25	38	1.25	1.84	0.67	0.65	996	1131
Σ ♂	Σ 6057	Σ 5984	Σ 194	Σ 109	3.20 ±1.97	1.83 ±0.27	0.53 ±0.13	0.52 ±0.14	Σ 5843	Σ 6216
Σ ♂ and ♀	Σ 12091	Σ 12039	Σ 279 ^B^	Σ 210 ^B^	2.30 ±1.66 ^B’^	1.75 ±0.18 ^B’^	0.54 ±0.06 ^C”^	0.66 ±0.16 ^C”^	Σ 11009	Σ 7961
*Doxorubicin hydrochloride – DXR (5 mg.Kg*^*–1*^*)*										
♀_1_	2091	2017	49	36	2.34	1.78	0.72	0.96	809	83
♀_2_	2106	2077	73	63	3.47	3.03	0.98	0.99	44	23
♀_3_	2056	2092	57	50	2.77	2.39	0.84	0.95	394	108
Σ ♀	Σ 6253	Σ 6186	Σ 179	Σ 149	2.86 ±0.57	2.40 ±0.62	0.85 ±0.13	0.97 ±0.02	Σ 1247	Σ 214
♂_1_	2067	2086	53	61	2.56	2.92	0.98	0.95	33	114
♂_2_	2063	2042	56	70	2.71	3.43	0.98	0.97	37	58
♂_3_	2082	2075	46	50	2.21	2.41	0.99	0.99	18	25
Σ ♂	Σ 6212	Σ 6203	Σ 155	Σ 181	2.50 ±0.26	2.92 ±0.51	0.99 ±0.00	0.97 ±0.02	Σ 88	Σ 197
Σ ♂ and ♀	Σ 12465	Σ 12389	Σ 334 ^C^	330 ^C^	2.68 ±0.42 ^C’^	2.66 ±0.43 ^C’^	0.92 ±0.07 ^A”^	0.97 ±0.01 ^A”^	Σ 1335	Σ 411
*POHALS – Pharmaceutical oil of H. annuus L. seeds (250 mg.Kg*^*–1*^*)*										
♀_1_	2081	2092	9	7	0.43	0.33	0.99	1.00	14	10
♀_2_	2086	2087	5	8	0.24	0.38	0.99	1.00	13	9
♀_3_	2090	2084	8	8	0.38	0.38	1.00	0.99	7	12
Σ ♀ ^A A^	Σ 6257	Σ 6263	Σ 22	Σ 23	0.35 ±0.10	0.37 ±0.03	0.99 ±0.00	1.00 ±0.00	Σ 34	Σ 31
♂_1_	2082	2083	10	16	0.48	0.77	0.99	0.99	18	11
♂_2_	2085	2099	7	9	0.34	0.43	0.99	1.00	15	9
♂_3_	2089	2072	9	15	0.43	0.72	0.99	0.99	11	21
Σ ♂ ^B A^	Σ 6256	Σ 6254	Σ 26	Σ 40	0.42 ±0.07	0.64 ±0.18	0.99 ±0.00	0.99 ±0.00	Σ 44	Σ 41
Σ ♂ and ♀	Σ 12513	Σ 12517	Σ 48 ^A^	Σ 63 ^A^	0.38 ±0.09 ^A’^	0.50 ±0.19 ^A’^	0.99 ±0.00 ^A”^	0.99 ±0.00 ^A”^	Σ 78	Σ 72
*POHALS – Pharmaceutical oil of H. annuus L. seeds (500 mg.Kg*^*–1*^*)*										
♀_1_	2021	2075	6	6	0.30	0.29	1.00	0.99	9	13
♀_2_	2047	2087	9	11	0.44	0.53	0.99	1.00	11	8
♀_3_	2034	2089	8	7	0.39	0.34	0.99	0.99	14	11
Σ ♀ ^A A^	Σ 6102	Σ 6251	Σ 23	Σ 24	0.38 ±0.07	0.38 ±0.13	0.99 ±0.00	0.99 ±0.00	Σ 34	Σ 32
♂_1_	2055	2057	10	11	0.49	0.53	0.99	0.99	28	18
♂_2_	2067	2071	18	15	0.87	0.72	0.99	0.99	11	16
♂_3_	2076	2082	16	19	0.77	0.91	1.00	0.99	7	12
Σ ♂ ^B A^	Σ 6198	Σ 6210	Σ 44	Σ 45	0.71 ±0.20	0.72 ±0.19	0.99 ±0.01	0.99 ±0.00	Σ 46	Σ 46
Σ ♂ and ♀	Σ 12300	Σ 12461	Σ 67 ^A^	Σ 69 ^A^	0.54 ±0.24 ^A’^	0.55 ±0.24 ^A’^	0.99 ±0.00 ^A”^	0.99 ±0.00 ^A”^	Σ 80	Σ 78
*POHALS – Pharmaceutical oil of H. annuus L. seeds (1,000 mg.Kg*^*–1*^*)*										
♀_1_	2088	2091	15	11	0.72	0.53	0.99	1.00	12	7
♀_2_	2084	2086	9	16	0.43	0.77	0.99	0.99	14	14
♀_3_	2090	2080	11	9	0.53	0.43	1.00	0.99	10	11
Σ ♀ ^A A^	Σ 6262	Σ 6257	Σ 35	Σ 36	0.56 ±0.15	0.58 ±0.17	0.99 ±0.00	0.99 ±0.00	Σ 36	Σ 32
♂_1_	2071	2077	14	23	0.68	1.11	0.99	0.99	18	18
♂_2_	2087	2093	21	15	1.01	0.72	0.99	0.99	13	11
♂_3_	2084	2079	17	15	0.82	0.72	0.99	0.99	16	17
Σ ♂ ^B A^	Σ 6242	Σ 6249	Σ 52	Σ 53	0.83 ±0.17	0.85 ±0.22	0.99 ±0.00	0.99 ±0.00	Σ 47	Σ 46
Σ ♂ and ♀	Σ 12504	Σ 12506	Σ 87 ^A^	Σ 89 ^A^	0.70 ±0.20 ^A’^	0.71 ±0.23 ^A’^	0.99 ±0.00 ^A”^	0.99 ±0.00 ^A”^	Σ 83	Σ 78
*POHALS – Pharmaceutical oil of H. annuus L. seeds (1,500 mg.Kg*^*–1*^*)*										
♀_1_	2091	2088	15	11	0.72	0.53	1.00	0.99	10	12
♀_2_	2091	2102	10	15	0.48	0.71	1.00	1.00	9	8
♀_3_	2084	2076	12	12	0.58	0.58	0.99	0.99	19	14
Σ ♀ ^A A^	Σ 6266	Σ 6266	Σ 37	Σ 38	0.59 ±0.12	0.61 ±0.10	0.99 ±0.00	0.99 ±0.00	Σ 38	Σ 34
♂_1_	2079	2084	21	20	1.01	0.96	0.99	0.99	21	16
♂_2_	2083	2067	18	17	0.86	0.82	0.99	0.99	19	21
♂_3_	2091	2085	17	21	0.81	1.01	0.99	0.99	18	15
Σ ♂ ^B A^	Σ 6253	Σ 6236	Σ 56	Σ 58	0.90 ±0.10	0.93 ±0.10	0.99 ±0.00	0.99 ±0.00	Σ 58	Σ 52
Σ ♂ and ♀	Σ 12519	Σ 12502	Σ 93 ^A^	Σ 96 ^A^	0.74 ±0.19 ^A’^	0.77 ±0.20 ^A’^	0.99 ±0.00 ^A”^	0.99 ±0.00 ^A”^	Σ 96	Σ 86
*POHALS – Pharmaceutical oil of H. annuus L. seeds (2,000 mg.kg*^*–1*^*)*										
♀_1_	2084	2091	18	17	0.86	0.81	0.99	1.00	17	9
♀_2_	2089	2087	16	18	0.77	0.86	0.99	0.99	15	13
♀_3_	2091	2085	9	13	0.43	0.62	0.99	0.99	11	15
Σ ♀ ^A A^	Σ 6264	Σ 6263	Σ 43	Σ 48	0.69 ±0.23	0.77 ±0.13	0.99 ±0.00	0.99 ±0.00	Σ 43	Σ 37
♂_1_	2071	2074	24	25	1.16	1.21	0.98	0.98	36	32
♂_2_	2085	2086	18	19	0.86	0.91	0.98	0.99	33	28
♂_3_	2048	2078	15	18	0.73	0.87	0.99	0.99	27	30
Σ ♂ ^B A^	Σ 6204	Σ 6238	Σ 57	Σ 62	0.92 ±0.22	0.99 ±0.18	0.98 ±0.00	0.99 ±0.00	Σ 96	Σ 90
Σ ♂ and ♀	Σ 12468	Σ 12501	Σ 100 ^A^	Σ 110 ^A^	0.80 ±0.24 ^A’^	0.88 ±0.19 ^A’^	0.99 ±0.00 ^A”^	0.99 ±0.00 ^A”^	Σ 139	Σ 127
*POHALS (2 g.kg*^*–1*^*) + NEU (50 mg.Kg*^*–1*^*)*										
♀_1_	2040	2054	67	51	3.28	2.48	0.73	0.68	760	946
♀_2_	2042	2068	61	61	2.99	2.95	0.73	0.65	758	1132
♀_3_	2039	2007	54	64	2.65	3.19	0.70	0.69	861	893
Σ ♀ ^A A^	Σ 6121	Σ 6129	Σ 182	Σ 176	2.97 ±0.32	2.87 ±0.36	0.72 ±0.01	0.67 ±0.02	Σ 2379	Σ 2971
♂_1_	2038	2014	46	52	2.26	2.58	0.64	0.69	1162	886
♂_2_	2011	2072	54	59	2.69	2.85	0.67	0.80	989	528
♂_3_	2008	2053	49	49	2.44	2.39	0.69	0.76	892	647
Σ ♂ ^B A^	Σ 6057	Σ 6139	Σ 149	Σ 160	2.46 ±0.21	2.61 ±0.23	0.67 ±0.03	0.75 ±0.05	Σ 3043	Σ 2061
Σ ♂ and ♀	Σ 12178	Σ 12268	Σ 331 ^C^	Σ 336 ^C^	2.72 ±0.37 ^C’^	2.74 ±0.31 ^C’^	0.69 ±0.04 ^B”^	0.71 ±0.06 ^B”^	Σ 5422	Σ 5032
*POHALS (2 g.kg*^*–1*^*) + DXR (5 mg.Kg*^*–1*^*)*										
♀_1_	2034	2166	76	52	3.74	2.40	0.64	0.83	1166	434
♀_2_	2069	2015	51	49	2.46	2.43	0.69	0.69	931	885
♀_3_	2017	2066	52	59	2.58	2.86	0.70	0.69	883	934
Σ ♀ ^A A^	Σ 6120	Σ 6247	Σ 179	Σ 160	2.93 ±0.70	2.56 ±0.25	0.67 ±0.03	0.74 ±0.08	Σ 2980	Σ 2253
♂_1_	2057	2017	53	55	2.58	2.73	0.66	0.78	1043	583
♂_2_	2056	2037	73	51	3.55	2.50	0.59	0.75	1444	663
♂_3_	2081	2021	47	49	2.26	2.42	0.74	0.78	719	579
Σ ♂ ^B A^	Σ 6194	Σ 6075	Σ 173	Σ 155	2.80 ±0.67	2.55 ±0.16	0.66 ±0.08	0.77 ±0.01	Σ 3206	Σ 1825
Σ ♂ and ♀	Σ 12314	Σ 12322	Σ 352 ^C^	Σ 315 ^C^	2.86 ±0.62 ^C’^	2.56 ±0.19 ^C’^	0.67 ±0.05 ^B”^	0.75 ±0.05 ^B”^	Σ 6186	Σ 4078

Means with the same letter are not significantly different (*p* < 0.05).

Shown are data from the controls (NaCl, NEU and DXR), an evaluation of the genotoxicity of POHALS, and an evaluation of the antigenotoxicity of POHALS (POHALS + NEU and POHALS + DXR).

**Table 3:** **The incidence of MNPCEs and PCE/NCE ratio in bone marrow of male and female *Swiss albinus *** **mice after testing for 24 h and 48 h**

**Treatment**	**Number of PCEs analyzed**		**PCEMNs**				**PCE/(PCE + NCE)**		**NCE ( *n *)**	
	**24 h**	**48 h**	**24 h**	**48 h**	**24 h**	**48 h**	**24 h **^**A”**^	**48 h **^**A”**^	**24 h**	**48 h**
			**( *n *) **^**A**^	**( *n *) **^**A**^	**(%) **^**A’**^	**(%) **^**A’**^				
*150 mM NaCl*										
♀_1_	2095	2097	7	10	0.33	0.48	1.00	1.00	5	3
♀_2_	2094	2095	9	10	0.43	0.48	1.00	1.00	6	5
♀_3_	2087	2089	11	8	0.53	0.38	0.99	0.99	13	11
Σ ♀	Σ 6276	Σ 6281	Σ 27	Σ 28	0.43 ±0.10	0.45 ±0.05	1.00 ±0.00	1.00 ±0.00	Σ 24	Σ 19
♂_1_	2095	2088	9	13	0.43	0.62	1.00	0.99	5	12
♂_2_	2055	2088	12	11	0.58	0.53	0.98	0.99	45	12
♂_3_	2058	2084	7	11	0.34	0.53	0.98	0.99	42	16
Σ ♂	Σ 6208	Σ 6260	Σ 28	Σ 35	0.45 ±0.12	0.56 ±0.06	0.99 ±0.01	0.99 ± 0.00	Σ 92	Σ 40
Σ ♂ and ♀	Σ 12484	Σ 12541	Σ 55 ^A^	Σ 63 ^A^	0.44 ±0.08 ^A’^	0.50 ±0.06 ^A’^	0.99 ±0.01 ^A”^	1.00 ±0.00 ^A”^	Σ 116	Σ 59
*N–Nitroso–N–ethylurea – NEU (50 mg.Kg*^*-1*^*)*										
♀_1_	2148	2075	38	36	1.77	1.73	0.49	0.65	2252	1125
♀_2_	1884	2032	32	34	1.70	1.67	0.54	0.81	1616	468
♀_3_	2002	1948	15	31	0.75	1.59	0.61	0.93	1298	152
Σ ♀	Σ 6034	Σ 6055	Σ 85	Σ 101	1.41 ±0.57	1.67 ±0.07	0.54 ±0.06	0.80 ±0.14	Σ 5166	Σ 1745
♂_1_	2025	1999	64	31	3.16	1.55	0.41	0.36	2875	3501
♂_2_	2028	1916	105	40	5.18	2.09	0.51	0.55	1972	1584
♂_3_	2004	2069	25	38	1.25	1.84	0.67	0.65	996	1131
Σ ♂	Σ 6057	Σ 5984	Σ 194	Σ 109	3.20 ±1.97	1.83 ±0.27	0.53 ±0.13	0.52 ±0.14	Σ 5843	Σ 6216
Σ ♂ and ♀	Σ 12091	Σ 12039	Σ 279 ^B^	Σ 210 ^B^	2.30 ±1.66 ^B’^	1.75 ±0.18 ^B’^	0.54 ±0.06 ^D”^	0.66 ±0.16 ^D”^	Σ 11009	Σ 7961
*Doxorubicin hydrochloride – DXR (5 mg.Kg*^*-1*^*)*										
♀_1_	2091	2017	49	36	2.34	1.78	0.72	0.96	809	83
♀_2_	2106	2077	73	63	3.47	3.03	0.98	0.99	44	23
♀_3_	2056	2092	57	50	2.77	2.39	0.84	0.95	394	108
Σ ♀	Σ 6253	Σ 6186	Σ 179	Σ 149	2.86 ±0.57	2.40 ±0.62	0.85 ±0.13	0.97 ±0.02	Σ 1247	Σ 214
♂_1_	2067	2086	53	61	2.56	2.92	0.98	0.95	33	114
♂_2_	2063	2042	56	70	2.71	3.43	0.98	0.97	37	58
♂_3_	2082	2075	46	50	2.21	2.41	0.99	0.99	18	25
Σ ♂	Σ 6212	Σ 6203	Σ 155	Σ 181	2.50 ±0.26	2.92 ±0.51	0.99 ±0.00	0.97 ±0.02	Σ 88	Σ 197
Σ ♂ and ♀	Σ 12465	Σ 12389	Σ 334^C^	330^C^	2.68 ±0.42 ^C’^	2.66 ±0.43 ^C’^	0.92 ±0.07 ^AB”^	0.97 ±0.01 ^AB”^	Σ 1335	Σ 411
*FOHALS – Food oil of H. annuus L. seeds (250 mg.Kg*^*-1*^*)*										
♀_1_	2137	2019	11	9	0.51	0.45	0.99	0.99	24	14
♀_2_	2142	2073	7	14	0.33	0.68	1.00	1.00	8	5
♀_3_	2146	2016	9	7	0.42	0.35	0.99	1.00	12	6
Σ ♀ ^A A^	Σ 6425	Σ 6108	Σ 27	Σ 30	0.42 ±0.09	0.49 ±0.17	0.99 ±0.00	1.00 ±0.00	Σ 44	Σ 25
♂_1_	2061	2079	6	10	0.29	0.48	0.95	0.94	98	140
♂_2_	2093	2093	8	7	0.38	0.33	0.99	0.99	31	20
♂_3_	2050	2041	8	6	0.39	0.29	0.99	0.97	30	59
Σ ♂ ^B B^	Σ 6204	Σ 6213	Σ 22	Σ 23	0.35 ±0.03	0.37 ±0.10	0.98 ±0.02	0.97 ±0.03	Σ 159	Σ 219
Σ ♂ and ♀	Σ 12629	Σ 12321	Σ 49 ^A^	Σ 53 ^A^	0.39 ±0.08 ^A’^	0.43 ±0.14 ^A’^	0.98 ±0.02 ^A”^	0.98 ±0.02 ^A”^	Σ 203	Σ 244
*FOHALS – Food oil of H. annuus L. seeds (500 mg.Kg*^*-1*^*)*										
♀_1_	2014	2046	10	11	0.50	0.54	0.98	0.99	39	24
♀_2_	2007	2094	8	8	0.40	0.38	0.95	0.98	106	34
♀_3_	2010	2035	9	16	0.45	0.79	0.96	0.99	81	11
Σ ♀ ^A A^	Σ 6031	Σ 6175	Σ 27	Σ 35	0.45 ±0.05	0.57 ±0.20	0.96 ±0.02	0.99 ±0.01	Σ 226	Σ 69
♂_1_	2037	2082	10	10	0.49	0.48	0.95	0.94	116	135
♂_2_	2063	2067	8	16	0.39	0.77	0.96	0.96	78	89
♂_3_	2053	2078	5	9	0.24	0.43	0.95	0.97	102	63
Σ ♂ ^B B^	Σ 6153	Σ 6227	Σ 23	Σ 35	0.37 ±0.12	0.56 ±0.18	0.95 ±0.01	0.96 ±0.02	Σ 296	Σ 287
Σ ♂ and ♀	Σ 12184	Σ 12402	Σ 50 ^A^	Σ 70 ^A^	0.41 ±0.09 ^A’^	0.57 ±0.17 ^A’^	0.96 ±0.01 ^AB”^	0.97 ±0.02 ^AB”^	Σ 522	Σ 356
*FOHALS – Food oil of H. annuus L. seeds (1,000 mg.Kg*^*-1*^*)*										
♀_1_	2114	2083	11	16	0.52	0.77	0.98	0.98	36	48
♀_2_	2148	2058	11	13	0.51	0.63	0.96	0.97	84	72
♀_3_	2097	2090	8	9	0.38	0.43	0.93	0.95	149	106
Σ ♀ ^A A^	Σ 6359	Σ 6231	Σ 30	Σ 38	0.47 ±0.08	0.61 ±0.17	0.96 ±0.02	0.97 ±0.01	Σ 269	Σ 226
♂_1_	2073	2026	9	17	0.43	0.84	0.90	0.96	227	74
♂_2_	2065	2071	12	13	0.58	0.63	0.99	0.94	20	129
♂_3_	2003	2084	5	8	0.25	0.38	0.95	0.91	97	207
Σ ♂ ^B B^	Σ 6141	Σ 6181	Σ 26	Σ 38	0.42 ±0.17	0.62 ±0.23	0.95 ±0.02	0.94 ±0.03	Σ 344	Σ 410
Σ ♂ and ♀	Σ 12500	Σ 12412	Σ 56 ^A^	Σ 76 ^A^	0.45 ±0.12 ^A’^	0.61 ±0.18 ^A’^	0.95 ±0.03 ^AB”^	0.95 ±0.02 ^AB”^	Σ 613	Σ 636
*FOHALS – Food oil of H. annuus L. seeds (1,500 mg.Kg*^*-1*^*)*										
♀_1_	2065	2021	6	15	0.29	0.74	0.98	0.90	35	236
♀_2_	2041	2081	13	10	0.64	0.48	0.93	0.89	159	262
♀_3_	2068	2072	11	14	0.53	0.68	0.89	0.88	247	294
Σ ♀ ^A A^	Σ 6174	Σ 6174	Σ 30	Σ 39	0.49 ±0.18	0.63 ±0.14	0.93 ±0.05	0.89 ±0.01	Σ 441	Σ 792
♂_1_	2106	2087	10	18	0.47	0.86	0.92	0.96	194	94
♂_2_	2011	2088	5	11	0.25	0.53	0.91	0.94	189	126
♂_3_	2039	2128	14	16	0.69	0.75	0.98	0.88	38	277
Σ ♂ ^B B^	Σ 6156	Σ 6303	Σ 29	Σ 45	0.47 ±0.22	0.71 ±0.17	0.94 ±0.04	0.93 ±0.04	Σ 421	Σ 497
Σ ♂ and ♀	Σ 12330	Σ 12477	Σ 59 ^A^	Σ 84 ^A^	0.48 ±0.18 ^A’^	0.67 ±0.15 ^A’^	0.94 ±0.04 ^AB”^	0.91 ±0.03 ^AB”^	Σ 862	Σ 1289
*FOHALS – Food oil of H. annuus L. seeds (2,000 mg.kg*^*-1*^*)*										
♀_1_	2084	2015	11	13	0.53	0.65	0.89	0.86	245	325
♀_2_	2096	2025	13	11	0.62	0.54	0.91	0.83	216	407
♀_3_	2076	2002	9	17	0.43	0.85	0.95	0.94	114	126
Σ ♀ ^A A^	Σ 6256	Σ 6042	Σ 33	Σ 41	0.53 ±0.09	0.68 ±0.16	0.92 ±0.03	0.88 ±0.06	Σ 575	Σ 858
♂_1_	2057	2199	16	20	0.78	0.91	0.94	0.97	143	65
♂_2_	2016	2158	17	12	0.84	0.56	0.95	0.86	110	347
♂_3_	2106	2133	11	15	0.52	0.70	0.92	0.89	194	261
Σ ♂ ^B B^	Σ 6179	Σ 6490	Σ 44	Σ 47	0.71 ±0.17	0.72 ±0.18	0.93 ±0.02	0.91 ±0.06	Σ 447	Σ 673
Σ ♂ and ♀	Σ 12435	Σ 12532	Σ 77 ^A^	Σ 88 ^A^	0.62 ±0.16 ^A’^	0.70 ±0.15 ^A’^	0.92 ±0.02 ^B”^	0.89 ±0.05 ^B”^	Σ 1022	Σ 1531
*FOHALS (2 g.kg*^*-1*^*) + NEU (50 mg.Kg*^*-1*^*)*										
♀_1_	2020	2062	54	49	2.67	2.38	0.70	0.74	880	738
♀_2_	2080	2052	44	52	2.12	2.53	0.80	0.68	520	948
♀_3_	2008	2011	50	54	2.49	2.69	0.69	0.74	892	689
Σ ♀ ^A A^	Σ 6108	Σ 6125	Σ 148	Σ 155	2.43 ±0.28	2.53 ±0.15	0.73 ±0.06	0.72 ±0.03	Σ 2292	Σ 2375
♂_1_	2031	2013	50	67	2.46	3.33	0.73	0.75	769	687
♂_2_	2010	2054	43	67	2.14	3.26	0.65	0.76	1090	646
♂_3_	2042	2045	56	71	2.74	3.47	0.64	0.71	1158	855
Σ ♂ ^B B^	Σ 6083	Σ 6112	Σ 149	Σ 205	2.45 ±0.30	3.35 ±0.11	0.67 ±0.05	0.74 ±0.03	Σ 3017	Σ 2188
Σ ♂ and ♀	Σ 12191	Σ 12237	Σ 297^C^	Σ 360^C^	2.44 ±0.26 ^C’^	2.94 ±0.47 ^C’^	0.70 ±0.06 ^C”^	0.73 ±0.03 ^C”^	Σ 5309	Σ 4563
*FOHALS (2 g.kg*^*-1*^*) + DXR (5 mg.Kg*^*-1*^*)*										
♀_1_	2007	2051	50	54	2.49	2.63	0.77	0.82	593	449
♀_2_	2010	2033	64	49	3.18	2.41	0.77	0.88	590	267
♀_3_	2094	2031	51	54	2.44	2.66	0.84	0.85	406	369
Σ ♀ ^A A^	Σ 6111	Σ 6115	Σ 165	Σ 157	2.70 ±0.42	2.57 ±0.14	0.79 ±0.04	0.85 ±0.03	Σ 1589	Σ 1085
♂_1_	2128	2067	67	66	3.15	3.19	0.85	0.67	372	1033
♂_2_	2015	2053	79	65	3.92	3.17	0.69	0.71	885	847
♂_3_	2054	2041	66	60	3.21	2.94	0.64	0.66	1146	1059
Σ ♂ ^B B^	Σ 6197	Σ 6161	Σ 212	Σ 191	3.43 ±0.43	3.10 ±0.14	0.73 ±0.11	0.68 ±0.03	Σ 2403	Σ 2939
Σ ♂ and ♀	Σ 12308	Σ 12276	Σ 377^C^	Σ 348^C^	3.07 ±0.55 ^C’^	2.83 ±0.32 ^C’^	0.76 ±0.08 ^C”^	0.76 ±0.10 ^C”^	Σ 3992	Σ 4024

Means with the same letter are not significantly different (*p* < 0.05).

Shown are data from the controls (NaCl, NEU and DXR), an evaluation of the genotoxicity of FOHALS, and an evaluation of the antigenotoxicity of FOHALS (FOHALS + NEU and FOHALS + DXR).

For the first time, this research has provided information on the genotoxic and antigenotoxic effects of THALS. However, genotoxic studies of sunflower oil and oil sunflower ozonized (at a dose limit of 2 g.kg^-1^.d^-1^, based on evidence of toxicity from subchronic studies via intragastric administration of the product) were previously carried out using the MN assay in the bone marrow of mice using male and female Cenp: NMRI mice [31]. In this study, the treatment with sunflower oil did not cause cytotoxic damage to erithrocytes, as reported in the analyses of the PCE/NCE ratio, which corroborate with our findings from the pharmaceutical oil and partially with food oil. Likewise, that research proposes the hypothesis that no clastogenic effect occurs in the bone marrow of animals treated with the sunflower oil under experimental conditions [31].

Other studies have investigated the suitability of different vegetable oils for the human diet, reporting reductions in genotoxicity and cancer potentiation by sesame oil [32] sunflower oil [33], perilla and palm oil [34], olive, sunflower, peanut, corn, and soy oils [35], flax seed oil [36], and coconut oil [37], among others. The possible role of fatty acids, a main component of vegetable oils, in modulating genotoxicity and carcinogenicity has also been studied. The genotoxic activity of vegetable oils [seed oils of sesame, sunflower, wheat germ, flax, and soy oil, and both first–class extra–virgin and low–grade (refined) olive oil] consumed by humans were also tested in a *Drosophila* somatic mutation and recombination test (the *Drosophila melanogaster* SMART assay) [30]. Flax oil produced the strongest response, while sesame, wheat germ, and soy oil showed some genotoxic activity. Sunflower oil and the low–grade olive oil gave inconclusive results or negative biological diagnoses, possibly due to lower concentrations of PUFAs, even as refined products, and extra–virgin olive oil was clearly not genotoxic. It has been argued that the genotoxicity of an oil is most likely due to the fatty acid composition of the oil, which after peroxidation can form specific DNA–adducts. Such results were in general agreement with evidence from experimental and epidemiological studies summarized by Bartsch and collaborators (1999) [38]: n–PUFAs are related to the generation of oxidative DNA damage, a high intake of n–6 PUFAs is implicated in some types of cancers, and n–9 MUFAs and n–3 PUFAs may have a role in cancer prevention. Additionally, it was suggested that the relative concentrations of short–chain C18:3 n–3 linolenic acid, C18:2 n–6 linoleic acid, and polyphenols are the major factors responsible for the genotoxicity of cooking oils in the SMART assay [30]. Despite the existence of this information, contradictory or inconclusive data were found in the literature. For instance, one study reported that linoleic acid (C18:2 n–6 PUFA) suppressed cancer cell proliferation [39], while other studies indicated an enhancing effect on carcinogenesis [40,41]. Oleic acid (C18:1, n–9 MUFA), a promoter of cancer cell proliferation [39], has also been reported to be an effective anticancer and antigenotoxic agent [42,43]. Linolenic acid (C18:3 short–chain n–3 PUFA) had anticancer activity in some studies [39,44], but promoted cancer in other studies [41,45]. Phenolic compounds, another important constituent of vegetable oils, are present in the unsaponifiable lipid phase. Phenolics are involved in both extra- and intracellular processes, inducing cytosolic detoxifying mechanisms, microsomal enzyme activation, and the scavenging of free radicals [46,47]. Evidence indicates that polyphenols can inhibit the genotoxicity of genotoxic agents [48,49] and function as anticancer agents [50].

The clastogenic and cytotoxic effects from heated sunflower oil were studied in lymphocytes, hepatocytes (HepG2) and in human umbilical vein endothelial cells (HUVEC) [19]. In lymphocytes incubated with water extract of heated sunflower oil containing 0.075 or 0.15 μM of thiobarbituric acid–reactive substances (this extract has a high content in polar aldehydes), the rate of chromosomal breakage was 18.4% and 23.1%, compared to 8.7% and 6.6%, or 8.1% and 9.2%, respectively in lymphocytes incubated with the same volume of a water extract from non–heated oil or distilled water. In HepG2 or HUVEC cells, the cytotoxic properties of heated sunflower oil were dose dependent, and the cytotoxicity occurred at concentrations as low as 0.25 μM. In contrast, the same volume of non–heated oil or distilled water was non–toxic for these cells. The results show that a water extract obtained from heated oil is clastogenic and, in higher doses, cytotoxic. These data also suggested that a water extract, obtained from culinary oils submitted to heat stress, with a high content of aldehydes is clastogenic. It was speculated that the ingestion of large amounts of these products may also impact human health, especially in those diseases secondary to chromosomal breakage such as certain congenital malformations and certain types of cancer. This last fact can be corroborated by previous reports indicating that the administration of thermally stressed sunflower oil to rats is teratogenic [51].

Doxorubicin (DXR) is an important anthracyclines anticancer agent. It is a valuable component of various chemotherapeutic regimens for breast carcinoma and small-cell lung carcinoma. In metastatic thyroid carcinoma, DXR is most likely the best available agent [20]. However, DXR has been reported to induce micronuclei, chromatid and chromosome aberrations, and DNA single- and double-strand breaks *in vitro* and *in vivo*[52-56]. The genotoxicity of anticancer drugs is of special interest because of the risk of inducing secondary malignancies. Therefore, it is essential to screen for newer pharmacological agents that can protect the normal cells against DXR–induced cumulative (geno) toxicity. Many plants that have been widely used in traditional medicine are less toxic than pharmaceutical agents and have recently attracted the attention of researchers around the world. Plants contain many compounds, and it is likely that these can provide better protective effects than a single molecule [57]. The presence of many molecules in plants may be advantageous, as some of them may counteract the toxicity of others, and as a result, the net effect may be beneficial for therapeutic purposes. For example, the effect of various concentrations (200, 250, 300, 350, and 400 mg/kg body weight) of *Aegle marmelos* on the doxorubicin (DXR)–induced genotoxic effects in mice bone marrow was studied [20]. Treatment of mice with different concentrations of DXR (5, 10, or 15 mg.kg^-1^ body weight) resulted in a dose–dependent elevation in the frequency of micronucleated polychromatic and normochromatic erythrocytes in mouse bone marrow, and it was accompanied by a DXR dose–dependent decline in the PCE/NCE ratio. The treatment of mice with *Aegle marmelos*, orally once daily for 5 consecutive days before DXR treatment, significantly reduced the frequency of DXR–induced micronuclei and significant increased the PCE/NCE ratio at all scoring times. This observed chemoprotective effect may be due to the sum total of interaction between different ingredients of this complex mixture. The degree of protection may depend on the interaction of components individually or collectively with the genotoxic agent. The plausible mechanisms of action of *Aegle marmelos* in protecting against DXR–induced genomic insult were scavenging of O_2_^•–^ and ^•^OH and other free radicals, increase in antioxidant status, restoration of topoisomerase II activity, and inhibition of the formation of DXR–iron complex [20]. Another study was undertaken to evaluate the genotoxic potential of *Copaifera langsdorffii* Desf. leaf hydroalcoholic extract and its influence on the genotoxicity induced by chemotherapeutic agent DXR using the *Swiss* mouse peripheral blood micronucleus test. The results of this study demonstrated that *C. langsdorffii* Desf. was not itself genotoxic and that in animals treated with *C. langsdorffii* Desf. and DXR, the number of micronuclei was significantly decreased compared to animals receiving DXR alone. The putative antioxidant activity of one or more of the active compounds of *C. langsdorffii* Desf., including two major flavonoid heterosides (quercitrin and afzelin), may explain the effect of this plant on DXR genotoxicity [18].

## Conclusions

In conclusion, this research observed an absence of genotoxicity of a tincture and two oils of sunflower seeds, regardless of the dose tested and the treatment time (24–48 h), but sex-independent (sunflower tincture) or sex-dependent (sunflower oils). Antigenotoxic effects (anticlastogeny and/or antianeugeny) were observed using only a dose of the sunflower tincture in association with the chemotherapy agent DXR. Therefore, the sunflower tincture can promote a partial protection against the genotoxic effects induced by DXR. The sunflower tincture no showed systemic toxicity and it was dose- and time-independent and sex-dependent, whereas the systemic toxicity of sunflower oil can be dependent on its source and its highest dose used.

Other studies involving the genotoxicity and antigenotoxicity of *H. annuus* L. extracts and oils (seeds, flowers and leaves) should be conducted [including genotoxicity assays with *Salmonella typhimurium* test (Ames test) as an indicator of potential carcinogenicity to mammals, gene mutation test in mammalian cells (mouse lymphoma assay), cytogenetic and aneuploidy tests *in vitro*, micronucleus test in cultured cells *in vitro*, fluorescent *in situ* hybridization (FISH) test for mutagenesis, comet test to detect of DNA damage and repair in individual cells, and functional genomic and proteomic tests for mutagenesis (cDNA microarrays and other array analyses)], to characterize the potential effects and genotoxic and antigenotoxic mechanisms and, importantly, for the establishment of limits for human consumption, the delineation of potential risks to human health, and for rational strategies for implementing chemo-preventive measures.

## Competing interest

The authors have declared no competing interest.

## Authors’ contributions

MFGB, JEF and NMSO wrote and revised the draft, MCCR and TAS provided animals care and revised the draft, LSS and MRR aided micronucleus assays and revised the draft, MFGB and CTSD performed statistical analysis. MFGB and JEF have given final approval of the version to be published. All authors have read and approved the final manuscript.

## Pre-publication history

The pre-publication history for this paper can be accessed here:

http://www.biomedcentral.com/1472-6882/14/121/prepub
